# Localized electronic vacancy level and its effect on the properties of doped manganites

**DOI:** 10.1038/s41598-021-85945-5

**Published:** 2021-03-23

**Authors:** Dilson Juan, Miguel Pruneda, Valeria Ferrari

**Affiliations:** 1 Instituto Sabato, UNSAM - CNEA, Av. Gral Paz 1499, San Martín, 1650 Buenos Aires, Argentina; 2 Catalan Institute of Nanoscience and Nanotechnology - ICN2, CSIC and BIST, Campus UAB, 08193 Bellaterra, Spain; 3 Instituto de Nanociencia y Nanotecnología, CNEA - CONICET., Departamento de Física de la Materia Condensada, GIyA, CAC - CNEA., Av. Gral Paz 1499, San Martín, 1650 Buenos Aires, Argentina

**Keywords:** Electronic properties and materials, Electronic structure, Magnetic properties and materials

## Abstract

Oxygen vacancies are common to most metal oxides and usually play a crucial role in determining the properties of the host material. In this work, we perform *ab initio* calculations to study the influence of vacancies in doped manganites $\text {La}_{(1-\text {x})} \text {Sr}_{\text {x}} \text {MnO}_{3}$, varying both the vacancy concentration and the chemical composition within the ferromagnetic-metallic range ($0.2\,<\,\text {x}\,<\,0.5$). We find that oxygen vacancies give rise to a localized electronic level and analyse the effects that the possible occupation of this defect state can have on the physical properties of the host. In particular, we observe a substantial reduction of the exchange energy that favors spin-flipped configurations (local antiferromagnetism), which correlate with the weakening of the double-exchange interaction, the deterioration of the metallicity, and the degradation of ferromagnetism in reduced samples. In agreement with previous studies, vacancies give rise to a lattice expansion when the defect level is unoccupied. However, our calculations suggest that under low Sr concentrations the defect level can be populated, which conversely results in a local reduction of the lattice parameter. Although the exact energy position of this defect level is sensitive to the details of the electronic interactions, we argue that it is not far from the Fermi energy for optimally doped manganites ($\text {x}\,\sim \,1/3$), and thus its occupation could be tuned by controlling the number of available electrons, either with chemical doping or gating. Our results could have important implications for engineering the electronic properties of thin films in oxide compounds.

## Introduction

It is well known that oxygen vacancies in perovskite oxides have a great impact on the functional properties of these materials, modifying the interplay among lattice, charge, spin, and orbital degrees of freedom. In mixed-valence manganites, the coexistence of ferromagnetism and metallic conduction has been traditionally described via a combination of a strong electron-phonon interaction, and the double-exchange mechanism in which the Mn 3d electrons hop through the O 2p orbitals. An oxygen vacancy disrupts this mechanism, inducing structural, electronic and magnetic changes that could have dramatic consequences. A considerable number of studies, both experimental and theoretical works, have tried to identify these effects, mitigating the negative consequences that they could pose for applications, for example in spintronics^1,2^. In other cases, understanding the effect of oxygen vacancies is pursued to improve their positive consequences, such as for applications in catalysis, or solid oxide fuel cells^3^.

X-ray photoemission (XPS) and photoabsorption spectroscopies (XAS) on oxygen or manganese core-levels have been used to identify the presence of oxygen vacancies in samples grown under different oxygen environments, or subject to annealing treatments. Although O-1s photoemission and O-K edge XAS spectra show features that have been correlated to oxygen vacancy states, probably the most clear evidence for their presence comes from the Mn-L edge XAS spectra. In particular, a peak or shoulder at $\sim \,640\,\text {eV}$ has been attributed to the presence of $\text {Mn}^{+2}$, that is a deviation from the characteristic $\text {Mn}^{+3}/\text {Mn}^{+4}$ valence ratio^4^. It can be argued that the oxygen vacancy acts as electron donor, and the excess electron is localized in the proximity of the defect, giving rise to such $\text {Mn}^{+2}$ peak. Indeed, first principles DFT calculations have demonstrated the existence of a defect level in the minority spin gap^2,5–7^. The partial occupation of this level results in the lost of half-metallicity and has been linked to the degradation of the magnetic properties^5,6^. However, calculations show that the position of the defect level is very sensitive to structural relaxations^2,7^ and it is usually located above the Fermi level, close to the bottom of the conduction band, hence decreasing the minority spin-gap. Ultraviolet photoemission measurements also reveal a significant reduction of the work function in samples where the $\text {Mn}^{+2}$ peak is observed in XAS^8^, evidencing that the extra electrons left behind by the oxygen vacancy play an important role on the physical properties of the material.

The splitting of the Mn 3s XPS spectra is commonly used to estimate the oxidation state (and magnetic moment) of Mn atoms. In samples with significant presence of oxygen vacancies, an increase in the splitting was reported^7,9^ corresponding to a decrease in the formal valence of Mn ions. However, the opposite trend was found in samples annealed in UHV for an extended period of time (where oxygen vacancies are expected to develop), and not exposed to air^5^. It can be argued that under these conditions, the $\text {Mn}^{+2}$ state was not formed at the surface^5,10^, and that the XPS spectra reveals an increase of the bonding covalency. Interestingly, depopulation of the defect level in previous DFT calculations seems to be related to a weakening of the Mn bonding covalency and an increase of the Mn-Mn distance, which correlates nicely with a slight expansion of the lattice observed in X-ray diffraction experiments^7,11^.

In any case, these contradictory results might indicate that under certain circumstances the electronic defect level could be occupied, and under other conditions, the level is empty. The aim of this paper is to determine under what conditions could the defect state be occupied and to clarify what are the consequences in the materials properties. Using first principles calculations, we provide a description at the atomic level of the electronic, structural and magnetic properties of bulk $\text {La}_{(1-\text {x})} \text {Sr}_{\text {x}} \text {MnO}_{3}$ (LSMO) manganite with oxygen vacancies. We focus on the most studied and technologically relevant doping concentration, namely $\text {La}_{2/3} \text {Sr}_{1/3} \text {MnO}_{3}$, usually referred to as optimal-doping, mainly due to the fact that it has the highest Curie temperature $\sim \,370\,\text {K}$^12^. To study the influence of both global and local Sr doping, we work in the composition range $0.2\,<\,\text {x}\,<\,0.5$, where the metallic-ferromagnetic character is preserved, and we address the effect of vacancy concentration considering different sizes of supercell. In this way, we can tune the position of the Fermi energy and modify the occupation of the defect state, which could form a dispersive band for large concentration of vacancies due to defect-defect interactions. The role of electronic correlations is also reviewed, and a comparison with previous works is discussed.

By analysing the character of the vacancy electronic level along with the effect of structural parameters and the magnetic interactions, we connect our results with experimental evidences published in the literature. Our main findings can be summarized as: (i) oxygen vacancies give rise to a new electronic defect level that has its main contribution mostly from neighbouring Mn d orbitals pointing towards the vacant site; (ii) this defect level acts as a scattering center that might be partially responsible for the diminished transport properties observed in reduced LSMO samples; (iii) the occupation of this bonding level reduces the interatomic distance of the surrounding Mn leading to a local shrinking of the cell volume; (iv) conversely, depopulation of this defect level results in an increased interatomic distance leading to an overall volume expansion; (v) oxygen vacancies produce a weakening of the ferromagnetic interaction, as revealed by the substantial reduction of the exchange energy for the Mn ions close to the vacancy, regardless of the defect state occupation, and in agreement with the observed degradation of the magnetic properties of reduced LSMO.

## Results

### Pristine LSMO

**Figure 1:**
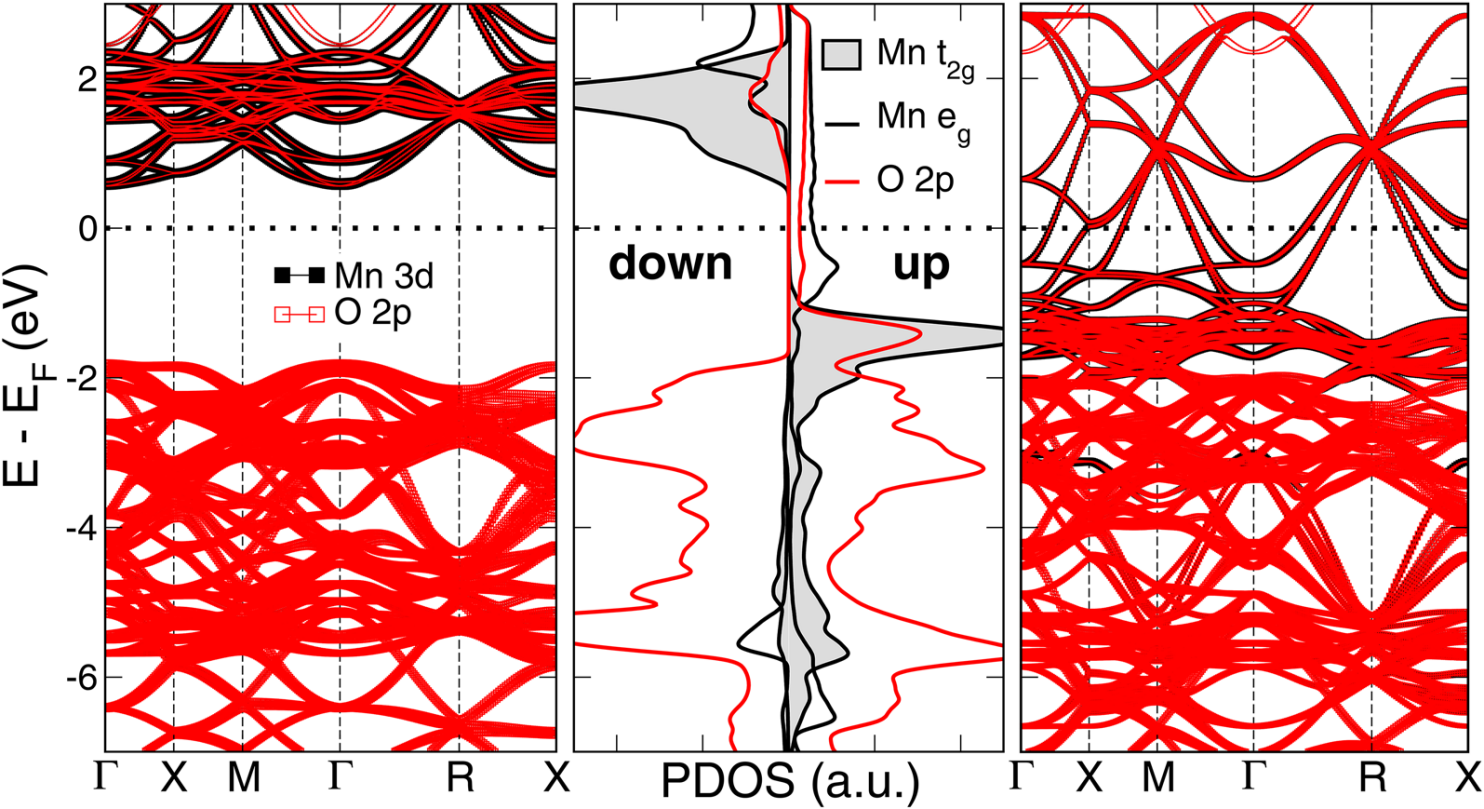
Electronic features of pristine LSMO. Left (Right) panels: Projected band structure for the spin down (up) channel for the Mn 3d and O 2p levels. Central panel: Partial Density of States (PDOS) highlighting the Mn $\text {t}_\text {2g}$ and the Mn $\text {e}_\text {g}$ contributions to the Mn 3d states (spin down/up corresponds to the left/right side of this panel, denoted by bold labels).

The $\text {La}_{(1-\text {x})} \text {Sr}_{\text {x}} \text {MnO}_{3}$ ($0.2\,<\,\text {x}\,<\,0.5$) crystal structure belongs to the $a^{-}a^{-}a^{-}$ Glazer’s tilt system^13^, which presents equal counterphase rotations of the octahedra around the three pseudocubic crystallographic axes. All the stoichiometric (pristine) supercells used in this study display this distortion pattern, with minimal dispersion of the octahedra rotations due to the direct substitution of the La cations for Sr dopant. Details can be found in the Supplementary Information (SI). The octahedral crystal field gives rise to the Mn 3d levels splitting into a lower energy triplet $\text {t}_{\text {2g}}$ and a higher energy $\text {e}_{\text {g}}$ doublet. Figure 1 plots the projected density of states (PDOS) and the orbital-projected Mn 3d and O 2p band structure for each spin channel. For the majority spin (up channel), the $\text {t}_{\text {2g}}$ level is completely filled while the $\text {e}_{\text {g}}$ level is just partially filled due to the holes introduced by the Sr doping. Therefore, the Mn 3d $\text {e}_{\text {g}}$ and O 2p levels extend around the Fermi level vicinity. This agrees with the fact that we do not observe any appreciable Jahn-Teller distortion of the octahedra^14^. For the minority spin (down channel), there is a pseudo-bandgap that decreases when increasing the Sr doping (see also Fig. S2 of SI online). The valence band maximum (VBM) is composed of O 2p orbitals while the conduction band minimum (CBM) has mainly empty Mn 3d $\text {t}_{\text {2g}}$ orbitals. Sr and La levels are very high in energy, well above the Fermi level. Consequently, within the range of doping considered in this work, the material presents half-metallicity with a 100% spin-polarization at the Fermi level^2,15^.

As the amount of Sr doping is increased (i.e. more holes are added), there is a rigid shift of the levels due to the Fermi energy moving downwards, but the half-metallic character of the system remains. Addition of a Hubbard term shifts the $\text {t}_{\text {2g}}^{\uparrow }$ levels towards lower energies and the $\text {t}_{\text {2g}}^{\downarrow }$ ones to higher energies. This results in an increased oxygen hybridization and delocalization of electronic levels, and a widening of the pseudo-bandgap with GGA + U, as compared to the bare GGA results. As expected, there is a minor underestimation of the pseudo-bandgap value ($\text {E}_{\text {g}}$) for bare GGA, and a better agreement with the experimentally reported gap^16^ when a value of $\text {U}\,\sim \,1\,\text {eV}$ is used (Fig. S2 in SI online). Our results also reproduce the strong ferromagnetic coupling resulting from the double-exchange Mn interaction mediated by oxygen atoms, as flipping the magnetization for any single Mn atom in this scenario has an energy cost of roughly 0.5 eV.

### Reduced LSMO: oxygen vacancies in the bulk

In this section we focus our study on the reduced host, namely $\text {La}_{(1-\text {x})} \text {Sr}_{\text {x}} \text {MnO}_{3-\delta }$, by introducing oxygen vacancies in a supercell structure. The size of the supercell determines the defect concentration, or the degree of reduction in the system ($\delta$). The diluted limit is studied by using a $2\times 2\times 6$ supercell with a low defect concentration ($\delta \,=\,1.4\,\%$), and the vacancy-rich limit is addressed with a $2\times 2\times 2$ supercell corresponding to a larger defect concentration ($\delta \,=\,4.2\,\%$). The oxygen vacancy formation energy ($\text {E}_{\text {for}}$) is calculated by evaluating the Kohn-Sham energy of the pristine system $\text {E}_{\text {pristine}}$, the reduced system $\text {E}_{\text {defect}}$ and the chemical potential of the removed oxygen atom, $\mu _{\text{ O }}$:

1 $$\begin{aligned} E_{\text {for}}\,=\,E_{\text {defect}}-E_{\text {pristine}}+\mu _{\text {O}} \end{aligned}$$

where the chemical potential for an oxygen atom is determined from the energy of the oxygen molecule in the gaseous phase, $\mu _{\text {O}}=1/2E_{\text {O}_2}$, thus assuming an oxygen-rich environment. Equation (1) is a simplification of a general expression extensively used in DFT approaches^17,18^ which is restricted in our case for neutral defects, because our system is metallic and the electronic reservoir is determined by the position of the Fermi level.

**Figure 2:**
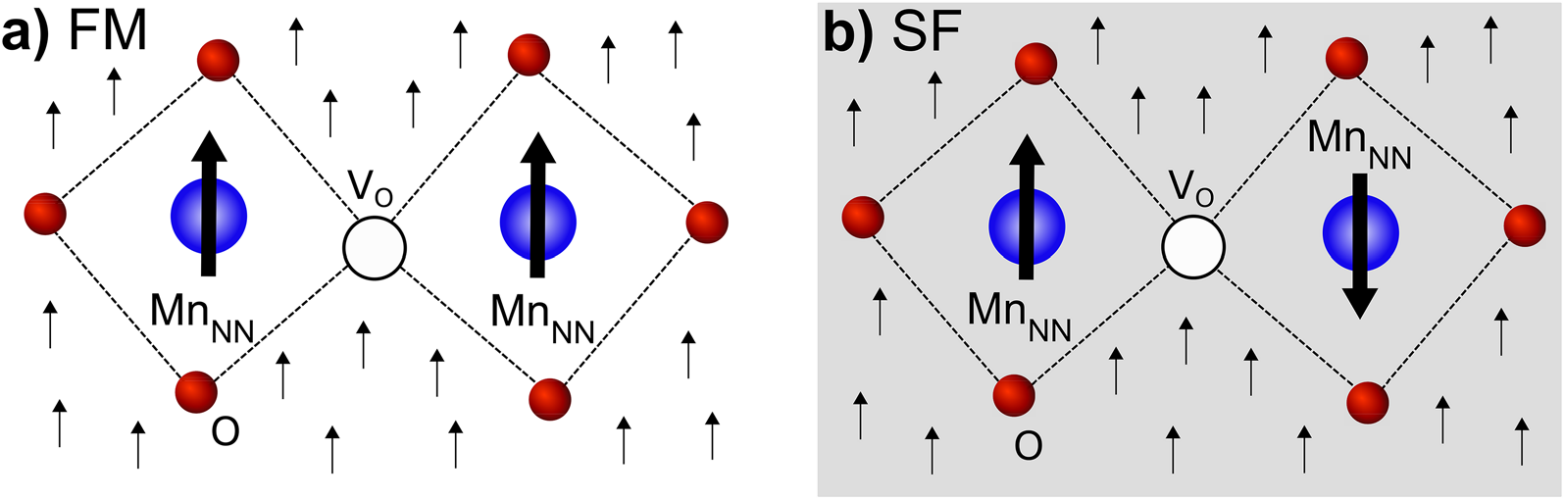
Schematics of the two magnetic configurations in the proximity of the vacancy. (**a**) Ferromagnetic state (FM). (**b**) Spin-flip state (SF). $\text {Mn}_{\text {NN}}$ (blue spheres) stands for the Mn atoms nearest neighbors to the oxygen vacancy ($\text {V}_{\text {O}}$, white sphere) and O (red spheres) represents oxygen atoms. The ferromagnetic host is represented by the upright arrows in the background.

The lack of an oxygen atom bridging two neighbors Mn ions reduces the electronic hopping behind the double-exchange ferromagnetic mechanism, and favors superexchange interactions that can result in anti-parallel magnetic configurations for the two Mn atoms linked to the vacancy. To explore this hypothesis, in the following we analyze two different magnetic states illustrated in Fig. 2: (i) the ferromagnetic configuration (which we name FM), where all Mn have parallel magnetic moments; and (ii) a local antiferromagnetic arrangement that involves a spin-flip process for one of the Mn atoms that is nearest neighbour to the vacancy (which we label as SF configuration).

#### Diluted limit

**Figure 3:**
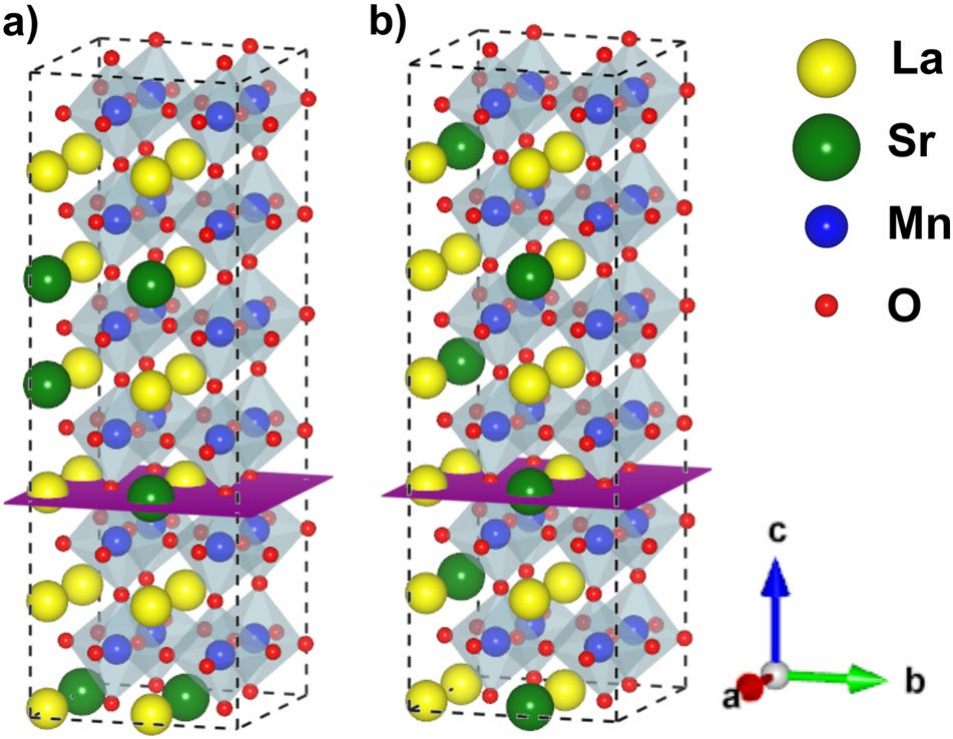
LSMO pseudocubic $2\times 2\times 6$ supercells employed. (**a**) $\text {x}\,=\,0.25$ and random doping, (**b**) $\text {x}\,=\,0.25$ and ordered doping. Medium size blue spheres represent Mn atoms, little red spheres are O atoms, big yellow spheres are La atoms and bigger green spheres, Sr atoms. The violet shaded area indicates the ab-plane where the oxygen vacancy is placed. Structures visualized with VESTA^19^.

To explore the limit corresponding to a diluted vacancy concentration, where vacancies are considered as isolated defects within the bulk of LSMO, we take a $2\times 2\times 6$ supercell with 120 atoms. This supercell is large enough to simulate a variety of Sr compositions and in addition, allows us to explore the effect of different local distributions of dopants (in particular, the effect of Sr clustering). With this purpose, we take two possible arrangements for the Sr atoms within the cell: (a) an ordered distribution were the Sr cations are uniformly dispersed across the bulk and local effects are minimized; and (b) a non-uniform distribution inspired on the concept of a random substitutional binary alloy, where the occupation probability of each A-site is given by the composition weight of the element. We refer to the latter as random distribution. Figure 3 illustrates two examples of the supercells used. Regarding oxygen vacancy concentration, we study the diluted limit with a reduction of $\delta \,=\,1.4\,\%$ ($\text {La}_{(1-\text {x})} \text {Sr}_{\text {x}} \text {MnO}_{2.958}$). We verified, by placing one oxygen vacancy on the LaSrO ab-planes of the supercell (highlighted in Fig. 3), that the interaction between periodic image replicas is negligible (this is not the case when the vacancy is introduced in a $\text {MnO}_2$ plane). Besides, it is also reasonable to assume that a very low concentration of defects (diluted limit) will only affect the structure locally, and no significant changes in the lattice constants should happen. Therefore, in this limit we allow relaxation of the internal atomic coordinates, without modification on the lattice cell parameters (our relaxed geometries have values of internal stress lower than 0.8 GPa).

**Figure 4:**
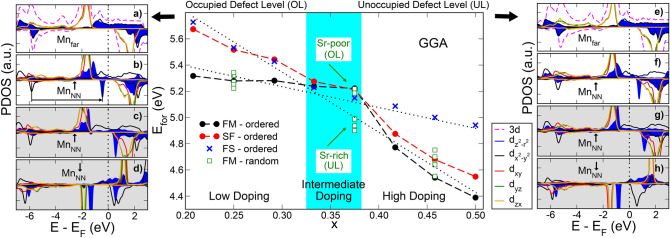
Left/Right Panel: PDOS on the Mn d orbitals for the low/high doping concentration. $\text {Mn}_{\text {far}}$ stands for a Mn atom located far away from the defect and $\text {Mn}_{\text {NN}}$ an atom nearest neighbour to the vacancy (see Fig. 2). The two topmost PDOS graphs are for the FM state while the two grey-shaded bottom graphs are for the spin-flip (SF) state. Dashed magenta line is the Mn 3d PDOS in bulk pristine LSMO. Central Panel: Formation energy for an isolated oxygen vacancy as a function of Sr doping (x), for the ordered and the random dopant distributions. For the ordered case, both FM and SF calculations are shown with dashed lines and the fix-spin (FS) case, with symbols.

When an oxygen vacancy is formed, the two electrons left behind are available for the bulk states. Consequently, there are visible modifications in the electronic structure, particularly in the levels of the Mn atoms surrounding the defect. This can be seen in Fig. 4 which compares the projected density of states (PDOS) for a reference bulk-like Mn atom (that is, a Mn atom located far from the defect, named as $\text {Mn}_{\text {far}}$) with the PDOS corresponding to the Mn atoms which are nearest neighbors (NN) to the oxygen vacancy, $\text {Mn}_{\text {NN}}$. The location of the oxygen vacancy is such that the Mn-$\text {V}_{\text{ O }}$-Mn chain is aligned along the z axis. Figure 4b corresponds to the FM case, where both $\text {Mn}_{\text {NN}}$ atoms are equivalent, thus only the PDOS corresponding to one of them is shown. The energy of the $\text {d}_{\text {z}^{2}-\text {r}^{2}}$ level, which in the pristine system overlaps with the oxygen 2p orbitals at − 6 eV, is now shifted upwards in energy by $\sim$ 5.5 eV, as schematically highlighted by the black arrow. Moreover, the contribution coming from the $\text {d}_{\text {z}^{2}-\text {r}^{2}}^{\uparrow }$ to the broad $\text {e}_\text {g}$ conduction band characteristic of LSMO half-metallicity in Fig. 4a, becomes more localized and forms a peak at about − 2 eV, with just a little tail crossing the Fermi energy in 4b. The $\text {d}_{\text {x}^{2}-\text {y}^{2}}$ on the other hand, remains mostly unaffected by the vacancy. Notice also that the removal of the oxygen atom transforms the symmetry of the system from octahedral to square pyramidal, thus lifting the degeneracy of the $\text {t}_{\text {2g}}$ levels.

There is, however, a feature that stands out when the vacancy is formed: a defect level in the minority spin channel. We argue that the position of this level relative to the Fermi energy critically determines the behaviour of the system. This defect state, can be either above or below the Fermi energy, thus changing its occupation depending on the Sr doping (i.e. the amount of holes added into the system). As a result, a slope change is observed in the formation energy as a function of Sr doping (central panel in Fig. 4), and three regions can be defined within the composition range analyzed: low, intermediate and high doping respectively, which we discuss in the following. Let us first address the case where the Sr distribution is homogeneous (ordered), and there is no local clustering.

At ***low doping*** concentration (equivalent to a few number of holes in the system) the defect level is occupied (OL) and the FM configuration is the ground state, as can be seen in the central panel of Fig. 4. The total magnetic moment in the unit cell does not change upon the formation of an oxygen vacancy, which is consistent with a picture where the two electrons left behind by the vacancy have opposite spins. The defect state has mostly s-type character, with bonding contribution from the neighbouring Mn $\text {d}_{\text {z}^{2}-\text {r}^{2}}$ orbitals. Consequently, these $\text {Mn}_{\text {NN}}$ ions shorten their interatomic distance and reduce their valence charge, as compared to the reference bulk values (see Tables 1 and 2, respectively). Although the reduced valence correlates well with the image of the $\text {Mn}^{+2}$ peak observed in XAS experiments and relates to the formation of vacancies close to the surface of the manganite^7,20^, the reduced Mn-Mn distance goes in the opposite direction to a lattice expansion reported for vacancy-rich samples^11,21^.

At ***high doping*** concentration, the increased number of holes introduced by the Sr dopant lowers the Fermi energy and the defect level becomes depopulated (UL). The electron that was localized in this state, is now distributed in the available electronic levels, which in this case are the $\text {e}_{\text {g}}^\uparrow$ ones. Hence, the extra spin is distributed among the bulk Mn ions, resulting in a slight increase (by about $0.13\,\mu _{B}$) of their magnetic moment, as can be seen in Table 2. As a consequence, the total magnetic moment of the system is increased by $+2\,\mu _{B}$ with respect to the pristine reference value. From a structural viewpoint, the depopulation of the (bonding) defect level increases the $\text {Mn}_{\text {NN}}$ interatomic distance, which is in agreement with the aforementioned volume expansion.

The above mentioned increase in the system magnetization induced by the defect seems counterintuitive with the degradation of the reported saturation magnetization^11^. To elucidate this issue, we explore spin-flip (SF) configurations for the neighbouring Mn atoms (Fig. 2b) to have an estimation of the strength of the ferromagnetic interactions. Although this SF configuration is less stable than the FM solution, their energy difference ($\lesssim \,0.2\,\text{eV}$) is significantly diminished with respect to the pristine LSMO ($\sim \,0.5\,\text {eV}$). Analysis of the PDOS for both $\text {Mn}_{\text {NN}}$ is shown in the shaded lateral panels in Fig. 4. The most remarkable feature is that now the defect level shifts to higher energies, falling above the Fermi level (both in the low and high doping regions). Otherwise, the PDOS for $\text {Mn}_{\text {NN}}^{\uparrow }$ resembles the FM state, with $\text {d}_{\text {z}^{2}-\text {r}^{2}}$ state shifting by $\sim \,6.5\,\text {eV}$ for the spin down towards higher energies. For $\text {Mn}_{\text {NN}}^{\downarrow }$ the image is very similar, except that the peaks are sharper possibly due to a reduced interaction with the host FM matrix.

Interestingly, as a function of Sr doping, the energy difference between the SF and the FM configurations decreases in the low doping region while increases in the high doping one. This defines an ***intermediate doping*** region, where the FM and SF configurations are almost degenerate in energy (central panel of Fig. 4). This region can also be identified by a change in the slope of the defect formation energy versus the Sr doping. It is worth mentioning that the so called *optimally-doped* manganite composition, $\text {La}_{2/3} \text {Sr}_{1/3} \text {MnO}_{3}$, falls within this intermediate region. Electronically, the Fermi level falls on top (or very close) to the defect state in the FM configuration and this could drive an electronic instability, favouring the spin flipping in one of the neighbour Mn atoms.

To tune the occupation of the defect level, we perform a *Fix Spin* (FS) calculation, by forcing a constrain over the total magnetic moment of the simulation cell. Taking the pristine LSMO magnetic moment as a reference, we impose a $+2\,\mu _{B}$ increase in the FM configuration ($\Delta \text {M}$ in Table 1), which forces the extra electrons from the vacancy to occupy majority spin states. In the low doping regime, this procedure artificially pushes the defect level in the minority spin above the Fermi level. The vacancy formation energy in this situation is shown in Fig. 4 with blue symbols, and is higher than the FM case. This excited state is metastable, and releasing the FS constrain on the magnetic moment provokes the system to come back to the FM ground state. We use the same approach in the high doping regime to induce the occupation of the defect level by fixing the total magnetic moment to the pristine value. Again, this results in higher metastable energy solutions (shown by blue crosses in the central panel of Fig. 4). In this way, using constrained and unconstrained calculations, we can set the defect level to be either occupied (OL) or unoccupied (UL) across the whole composition range as highlighted by the visual guidelines in Fig. 4 (dotted black lines).

In the following, we discuss the effect of ***Sr clustering*** in the system. To address how different local Sr environments influence the electronic and magnetic properties of bulk LSMO in the presence of oxygen vacancies, we take $2\times 2\times 6$ supercells with different disordered Sr distributions with an overall global composition ($\text {x}\,=\,0.250, 0.375$ and 0.458) corresponding to the low, intermediate and high doping regimes. For each of these supercells, we placed an isolated oxygen vacancy with different local Sr concentrations. For *low doping*, the defect level is always occupied and the local environment introduces a $\sim \,0.05\,\text {eV}$ dispersion in the formation energy (green squares in Fig. 4). For *high doping*, the defect level is always unoccupied, with slightly larger dispersion in the energies depending on the local Sr environment ($\sim \,0.07\,\text {eV}$). The *intermediate doping* regime shows a richer landscape, with the defect level being occupied when the vacancy is placed in a Sr-poor environment while it is unoccupied when located in a Sr-rich region. As a side note, the formation energies for these two scenarios match nicely the dotted lines in Fig. 4. These findings are threefold relevant because the Sr doping mainly used for applications in devices based on LSMO, matches our intermediate composition, where nearly degenerate magnetic configurations (FM and SF) arise in the defective system. Besides, this region constitutes a narrow frontier between occupied/unoccupied defect state and a slight composition variation might lean the scale towards one state or the other. In this respect, segregation of Sr at the surface of LSMO in thin films has been broadly reported^22–24^ and our results evidence how local cation distribution strongly influences both the electronic and magnetic properties.

**Figure 5:**
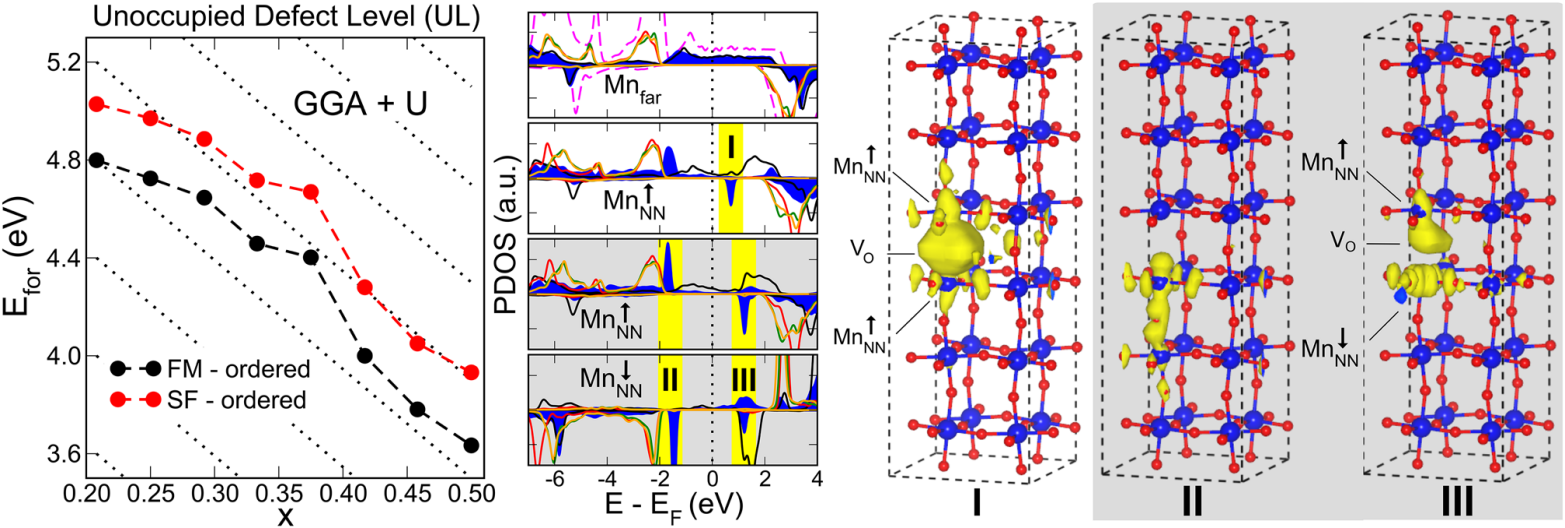
Left Panel: Formation energy for an isolated oxygen vacancy as a function of Sr doping for GGA + U calculation ($\text {U}\,=\,4.5\,\text {eV}$), with ordered Sr distribution. Dotted black lines as a grid depict the slope of UL for bare GGA. Central Panel: 3d Mn PDOS for low doping (same notation as in Fig. 4). Right Panel: Isosurfaces of electronic local density of states for the energy regions labelled I, II and III in the central panel. To highlight the defect level, only the spin down component is plotted.

At this point, we stress that our GGA treatment shows the presence of a defect level in the gap of the minority spin, which can be occupied or empty (below or above the Fermi level respectively), depending on the number of available electrons in the system. Although the presence of this defect level has been reported in previous $\textit{ab initio}$ calculations^2,5^, to our knowledge it has not been observed below the Fermi level and therefore, occupied. Most of the prior calculations include a Hubbard U term to account for strong electronic correlations. Even though our GGA results seem to give electronic features, particularly the energy position of Mn $\text {t}_{\text {2g}}$ peaks for the pristine system, that are in good agreement with experiments^25^, we also performed GGA + U calculations for the reduced case. Results obtained with a standard $\text {U}\,=\,4.5\,\text {eV}$ for all Mn 3d orbitals^26^ are presented in Fig. 5. In this case, the shift of the $\text {d}^{\downarrow }_{\text {z}^{2}-\text {r}^{2}}$ level increases to 6 eV, placing the defect state above the Fermi level for all the studied compositions. The $\text {3d}^{\uparrow \downarrow }$ exchange energy and the pseudo-bandgap, are also increased. The ground state is FM across the entire Sr range, and the SF configuration is consistently higher by $\sim \,0.25\,\text {eV}$. The electronic structure sketched by the PDOS on Mn nearest neighbors to the oxygen vacancy shares the same features described for the high doping regime using bare GGA, where the defect level is empty. The local density of states (LDOS) due to the defect state wavefunction is shown in the right panel of Fig. 5 where the bonding (antibonding) character for the FM (SF) configuration is evident.

#### Concentrated limit

**Figure 6:**
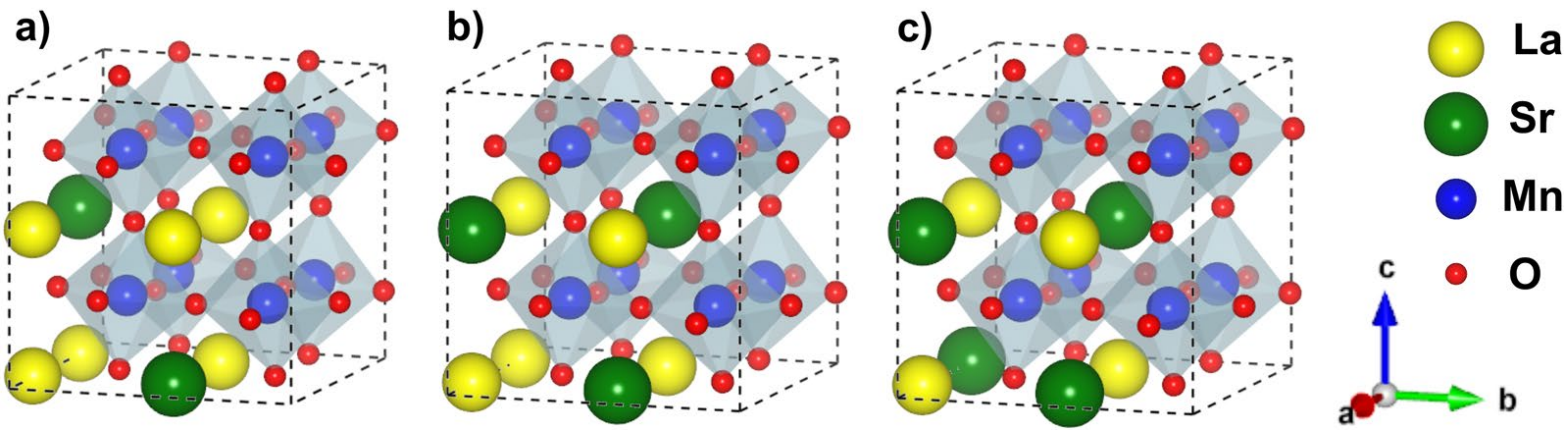
LSMO pseudocubic 2 × 2 × 2 supercells employed in the calculations. (**a**) $\text {x}\,=\,0.250$, (**b**) $\text {x}\,=\,0.375$, (**c**) $\text {x}\,=\,0.500$. Same notation as for Fig. 3.

When the number of oxygen vacancies increases, the interaction among the defect states can give rise to a dispersive band which can have an impact on the magnetic properties of the system. We explore this high vacancy concentration limit using a pseudocubic $2\times 2\times 2$ supercell with $R{{\overline{3}}}c$ symmetry that allows an adequate description of the octahedral distortions (Fig. 6). As before, Sr doping is modelled by direct substitution of La atoms by Sr ones. In this rather small supercell, clustering of the Sr atoms could result in artificially stacked heterostructures. Therefore, we adopt the so called anticlustering distribution, where the Sr atoms are disposed so that their mutual distance is maximized.

The removal of a single oxygen atom in this supercell corresponds to a $\delta \,=\,4.2\,\%$ reduction ($\text {La}_{0.825} \text {Sr}_{0.175} \text {MnO}_{2.875}$), where significant modifications to the lattice are expected to take place. Previous studies report a lattice expansion induced by the presence of oxygen vacancies in the system^11,27^. In particular, Guo et al.^7^ ascribed this expansion to the repulsion between the $\text {Mn}^{+3/+4}$ and $\text {La}^{+3}/\text {Sr}^{+2}$ cations which are nearest neighbors of the vacancy and the donor defect with a +2 formal charge. Here, we evaluate the effect on the geometry due to the Sr doping and the oxygen vacancies by allowing a full relaxation of both the atomic coordinates (with forces below 0.04 eV/Å), and the lattice parameters (with cell pressures smaller than 0.1 GPa). Our results agree with an expansion of the lattice parameter, except for the FM solution in the low Sr concentration regime, where GGA predicts an occupied defect state in the minority spin, and a contraction of the cell volume (see Fig. S2 of SI for more details). Notice that a local contraction of the Mn-Mn distance (Table 1) does not result in an overall volume contraction for the SF configuration for the same Sr concentration, which is slightly lower in energy than the FM phase. As we will discuss later, we suggest that this apparent dichotomy could actually explain some recent experimental observations.

The formation energy for oxygen vacancies in the concentrated limit is reported in Table 1 for both magnetic configurations analysed (FM and SF) and the corresponding doping concentration considered. We also include results obtained with the constrained-DFT approach (FS). The local distortion due to the vacancy defect is characterized by $\text {d}_{\text {Mn-Mn}}$, the interatomic distance between the two Mn atoms which are nearest neighbors to the vacant site. The difference in the total magnetic moment between the pristine and the defective system ($\Delta \text {M}$) is also reported in the table. As in the case of the diluted vacancy limit, the formation energy decreases as the Sr content increases. Furthermore, our data reveals that throughout the whole range of Sr compositions analyzed, the GGA ground state is the local spin-flip arrangement (SF). To the best of our knowledge, such a clear evidence of the weakening of the double-exchange mechanism in favor of superexchange antiferromagnetic interactions has not been reported before. This SF solution also results in a decreased magnetization of the system, and a slight shortening of $\text {d}_{\text {Mn-Mn}}$. On the contrary, under the GGA + U approximation, the FM becomes the most stable configuration with an unoccupied defect level, an augmented total magnetization in the defective system and an increased $\text {d}_{\text {Mn-Mn}}$.

**Table 1:** Summarized data for high vacancy concentration in LSMO with different Sr concentration (x): formation energy ($\text {E}_{\text {for}}$) with ground state energy highlighted in bold, interatomic distance between Mn nearest neighbors to the vacancy ($\text {d}_{\text {Mn-Mn}}$) and total magnetic moment change ($\Delta$M) between defective and pristine systems.

x		$\text {E}_{\text {for}}$		$\text {d}_{\text {Mn-Mn}}$ (NN)				$\Delta$M = $\text {M}_{\delta }$-M	
		(eV)		(Å)				($\mu _B$)	
		GGA	GGA + U	GGA		GGA + U		GGA	GGA + U
0.250	FM	5.42	**4.70**	3.88*	3.73	3.90*	3.95	+ 0	+ 2
	SF	**5.42**	4.75		3.84		3.92	− 6	− 6
	$\text {FS}^{\text {UL}}$	5.57	–		3.92		–	+ 2	–
0.375	FM	5.15	**4.34**	3.87*	3.86	3.88*	3.94	+ 0.9	+ 2
	SF	**5.03**	4.46		3.86		3.90	− 6	− 6
	$\text {FS}^{\text {OL}}$	5.27	–		3.79		–	+ 0	–
	$\text {FS}^{\text {UL}}$	5.18	–		3.93		–	+ 2	–
0.500	FM	4.83	**4.07**	3.86*	3.91	3.87*	3.93	+ 2	+ 2
	SF	**4.72**	4.20		3.82		3.85	− 6	− 6
	$\text {FS}^{\text {OL}}$	5.04	–		3.84		–	+ 0	–

GGA + U results correspond to $\text {U}\,=\,4.5\,\text {eV}$. As a reference, the average Mn-Mn distance for pristine LSMO is marked with an asterisk. Superscript OL (UL) stands for occupied (unoccupied) defect level.

Mulliken population analysis reinforces our interpretation of charge distribution throughout the studied cases. Table 2 collects the data of characteristic Mn atoms in the defective system for two specific Sr concentrations illustrating either the low and high doping regimes. For reference, Mulliken charges for the pristine ferromagnetic system are also presented. When the defect level in the FM state is occupied, the magnetic moment of neighbouring Mn atoms to the vacant site remains unaltered with respect to the pristine LSMO value, but the valence is reduced approaching the formal $\text {Mn}^{+3}$ value. This is a consequence of the localization of the extra electronic charge in a singlet state. The SF configuration shows a preferential increase in the net spin for the $\text {Mn}_{\text {NN}}^{\uparrow }$ atom that is aligned with the ferromagnetic host, and a decrease in the magnetic moment of the inverted $\text {Mn}_{\text {NN}}^{\downarrow }$. In the high doping regime (within bare GGA) or through the whole composition range upon the addition of the Hubbard term, the defect level becomes depopulated for the FM case and there is an increase in the magnetic moment of $0.13\,\mu _{B}$ in the $\text {Mn}_{\text {far}}$ and of $0.3{-}0.4\,\mu _{B}$ in both $\text {Mn}_{\text {NN}}^{\uparrow }$ with respect to the pristine system. Therefore in this case, the extra electronic charge left behind by the vacancy spreads over a wider region around the defect, and not just in the nearby Mn atoms.

**Table 2:** Mulliken population analysis for $\text {x}\,=\,0.25$, 0.50.

x		U	$\text {Mn}^{\text {LSMO}}$		$\text {Mn}_{\text {far}}$		$\text {Mn}_{\text {NN}}^{\uparrow }$		$\text {Mn}_{\text {NN}}^{\downarrow }$	
		(eV)	Q	M	Q	M	Q	M	Q	M
0.25	FM	0	1.70	3.60	1.70	3.63	0.99	3.64	–	–
		4.5	1.75	3.84	1.73	3.97	1.17	4.16	–	–
	SF	0	–	–	1.70	3.61	1.08	3.87	1.08	− 3.24
		4.5	–	–	1.74	3.84	1.15	4.08	1.18	− 3.62
0.50	FM	0	1.71	3.36	1.69	3.50	1.17	3.79	–	–
		4.5	1.76	3.62	1.76	3.76	1.22	4.01	–	–
	SF	0	–	–	1.71	3.50	1.12	3.73	1.12	− 3.35
		4.5	–	–	1.76	3.75	1.19	3.98	1.21	− 3.70

$\text {Mn}_{\text {NN}}$ are the nearest neighbors to the oxygen vacancy and $\text {Mn}_{\text {far}}$ refers to a Mn atom far away from the defect. $\text {Mn}^{\text {LSMO}}$ stands for a reference Mn atom of the pristine system. Q (e) represents the valence charge and M ($\mu _{\text {B}}$), the spin.

## Discussion and conclusions

In this work, first-principles calculations have been used to study the influence of oxygen vacancies in LSMO within the most typical range of compositions. In agreement with previous works^7^, we show that the creation of oxygen vacancies pushes the $\text {d}_{\text {z}^{2}-\text {r}^{2}}$ states from neighboring Mn atoms, towards higher energies. This results in a localized defect state whose energy falls within the gap of the minority spin component. We found that this defect level can be occupied, or at least partially occupied, under particular circumstances. This bonding state leads to: (i) a local accumulation of charge that could be linked to the $\text {Mn}^{+2}$ peak observed in XAS spectra^4^; (ii) a degradation of the average magnetization around the defect; (iii) an overall weakening of ferromagnetic interactions in the system and (iv) a scattering center that can be responsible for the degradation of the electronic conductivity in reduced systems.

Previous calculations were mostly based on a DFT + U approach, and showed no evidence that the defect state could be occupied^7,28^. When the Hubbard term is included in the Hamiltonian (with typical U values in the $3{-}5\,\text {eV}$ range, for all Mn 3d orbitals) the defect level is pushed towards higher energies and falls well above the Fermi level, for any Sr doping concentration. The extra charge left behind by the removed oxygen is distributed over the $\text {e}_{\text {g}}$ states, resulting in an increased net magnetization at odds with the degradation of the magnetization observed in experiments. The above results are based on the assumption that the U term is the same for all the Mn 3d orbitals, but the chemical environment close to the vacancy is different from that in the bulk, so there is no reason to support this approximation, and to assume that the defect state is always empty. Likewise, as we will discuss next, a few experimental observations in reduced LSMO samples can be better understood within an occupied defect level scenario.

The volume expansion measured by X-ray diffraction experiments^11^ is also found in our FM calculations (along with an increase of the Mn-Mn interatomic distance) when the defect level is empty, both in the GGA high doping limit and in GGA + U calculations. On the other hand, the bonding character of the defect state gives a reduced Mn-Mn distance when the level is occupied, leading to a small volume contraction, in opposition with the above-mentioned experimental findings. Although this might seem contradictory with our low doping bare GGA results, the DOS spectral features (including the pseudo-bandgap value) obtained with GGA are in better agreement with experimental measurements compared to GGA + U. Furthermore, LSMO thin films grown under low oxygen pressure (on top of $\text {SrTiO}_3$) with a Sr compositional gradient, recently revealed Sr-poor regions displaying a compression of the out of plane lattice parameter along with a complex magnetic behaviour^29^. Under these growth conditions, oxygen vacancies are expected throughout the sample and according to our predictions, the occupation of the bonding defect level is favoured, thus locally reducing the lattice parameter and inducing a softening of the ferromagnetic properties. In this way, our results might contribute to shed light on the mentioned contrasting observations in these monolithic films, which could not be explained when the defect level is empty.

A ferromagnetic metal to insulating transition was reported and related to the electromigration of mobile oxygen ions when electrons were added to doped manganites by means of electrolyte-gating^30^. In this regard, we found a weakening of the double-exchange mechanism revealed by the substantial reduction of the spin-flip energy configuration with respect to the pristine LSMO value. In the vacancy-diluted limit, the ground state remains ferromagnetic, but when the vacancy concentration increases, the superexchange interaction favors the SF arrangement over the FM one. It is expected that under a high vacancy concentration the interaction among defect levels gives rise to a dispersive defect band, increasing the range of Sr compositions for which the defect state could be occupied, thus destabilizing the ferromagnetic coupling. Previous calculations^28^ have shown that a gap is opened for an antiferromagnetic phase in bulk LSMO within the range of chemical compositions addressed here. All the above pieces could fit together considering a large concentration of vacancies along with the addition of electrons to populate the defect level. This procedure would weaken the ferromagnetic interactions, favoring antiferromagnetism and a bandgap opening.

Electrostatic gating in field-effect transistors^30,31^ could be used to tune the electronic chemical potential, thus changing the occupation of the defect state. In this regard, our GGA calculations predict that the optimal Sr doping concentration ($\text {x}\,=\,1/3$) belongs to the region where the Fermi level falls near the defect level, driving the electronic instability that favours spin flip arrangements. In this manner, small variations in the gate voltage could have dramatic effects on the electronic properties of reduced manganites, and we propose this experimental approach as a mean to validate the predictions of our calculations. We anticipate that the important insights revealed in this paper regarding the effects arising from an electronic defect state due to oxygen vacancies will improve our understanding of the LSMO physical properties and trigger further development of devices and applications.

## Methods

To perform total energy calculations we employ the SIESTA code^32^ within the GGA scheme, using the exchange-correlation functional WC^33^ (Wu-Cohen, modification of the PBE functional). Non local Troulliers-Martins pseudopotentials^34^ are employed, which conserve the norm and are factorized by the Kleinman-Bylander method^35^. All pseudopotentials and bases used in this work were developed and optimized in Ref.^25^. The pseudopotential of manganese was generated using the atomic configuration $3\text {s}^{2}3\text {p}^{6}3\text {d}^{5}5\text {f}^{0}$ with cutoff radii of 1.39, 1.88, 1.48 and 1.88 for the orbitals s, p, d and f respectively. In the case of oxygen, a configuration $2\text {s}^{2}2\text {p}^{4}3\text {d}^{0}4\text {f}^{0}$ was used with cutoff radii equal to 1.14 for each of the channels s, p, d and f. For strontium, the valence configuration for the pseudopotential was $4\text {s}^{2}4\text {p}^{6}$ (cutoff radius 1.49 for both cases) $4\text {d}^{0}4\text {f}^{0}$ (cutoff radius 1.99). The pseudopotential of lanthanum was generated using the atomic configuration $5\text {s}^{2}5\text {p}^{6}5\text {d}^{0}4\text {f}^{0}$ with cutoff radii 1.83, 2.19, 3.06 and 1.39 for the orbitals s, p, d and f respectively. For Mn, La and Sr relativistic effects were considered, but spin-orbit interactions were discarded. In these three elements, semicore states were also included. Finally, to model oxygen vacancies we employ the ghost atom feature included in SIESTA, considering the same pseudopotential and basis used for the oxygen atom. For the $2\times 2\times 2$ supercell, we take a 1600 Ry energy cutoff to perform the numerical integration in the real space and a $18\times 18\times 18$ k-points mesh in the Monkhorst-Pack scheme^36^, for the structural optimization. K-points mesh is reduced accordingly to $18\times 18\times 6$ for the $2\times 2\times 6$ supercell (with 120 atoms). For the density of states (DOS) calculation, we take a denser grid of k-points to obtain an accurate description of the energy levels. Convergence analysis were performed indicating less than 1.25 meV/u.f. energy error. The use of a computational methodology that accentuate Coulomb interactions among highly localized orbitals, such as DFT + U^37,38^ or hybrid functionals^39^ (HSE^40^, B3LYP^41^, PBE0^42^, etc) has been shown not necessary by previous studies in the composition range analyzed of pristine LSMO, characterized by a metallic state and Jahn-Teller dynamic distortions^25,43–45^. Regarding reduced LSMO and its defect state, it is fair to assume that bare DFT might underestimate the position of that level. Although a more complete description of the electronic interactions (such as hybrid functionals or beyond) might be needed to precisely determine the energy position of the defect level, these calculations are computationally challenging and exceed the scope of this work.

## Supplementary Information

Supplementary Information.

## Data Availability

All data generated or analysed during this study are included in this published article and its Supplementary Information files.
