# Supplementary material for: Localized electronic vacancy level and its effect on the properties of doped manganites
Source: Sci Rep. 2021 Mar 23;11:6706. doi: 10.1038/s41598-021-85945-5 (PMC7988069; doi:10.1038/s41598-021-85945-5)
Supplement: Supplementary file 1 — Supplementary Information. [file 41598_2021_85945_MOESM1_ESM.pdf]

Supplementary Information

Localized electronic vacancy level and its effect on the properties of doped manganites

Dilson Juan[1,2,3,*], Miguel Pruneda[2], and Valeria Ferrari[1,3]

[1]Instituto Sabato, UNSAM - CNEA, Av. Gral Paz 1499, San Martín, 1650 Buenos Aires, Argentina.
[2]Catalan Institute of Nanoscience and Nanotechnology - ICN2, CSIC and BIST, Campus UAB, 08193 Bellaterra, Spain.
[3]Instituto de Nanociencia y Nanotecnología, CNEA - CONICET. Departamento de Física de la Materia Condensada, GIyA, CAC - CNEA, Av. Gral Paz 1499, San Martín, 1650 Buenos Aires, Argentina.
[*]dilson.juan@icn2.cat

Pristine LSMO: structural and electronic properties

We have assessed how A-cation aliovalent substitution modify the lattice cell parameters by relaxing these degree of freedom in the supercells used in the calculations. The behaviour follows the empirical trend of volume cell decrease with increasing Sr content. This is shown in Fig. S1 that displays GGA and GGA + U (with U = 4.5 eV) results along with experimental values for comparison. The equilibrium lattice parameter presents a linear trend as a function of doping concentration. The relationship between the equilibrium lattice parameter and the value of U is shown in the inset of Fig. S1 for x = 0.375. Taking into account that there is an important dispersion in the different experimental values, we can not conclude that an unique U value can reproduce all the data. However, bare GGA or a Hubbard parameter of ~ 1.5 eV provide overall better description for the experimental values. Across the entire interval of composition studied we obtain an approximately 0.7 % lattice cell parameter variation. Deviations from the cubic ideal structure are small, with all the octahedra being regular for the whole doping range.

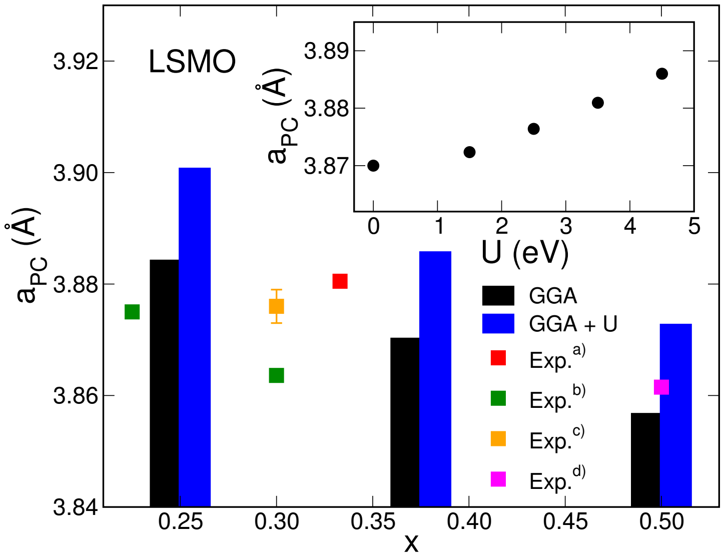

Figure S1. Lattice pseudocubic parameter (a_{PC}) as a function of Sr doping for pristine LSMO. Experimental data taken from: a) Ref.[1]; b) Ref.[2]; c) Ref.[3]; d) Ref.[4]. Inset: a_{PC} versus U for x = 0.375.

Other structural parameters were measured in the optimized structures. The Mn-O bonding distance is around 1.95 - 1.94 Å. The tilting angle of the octahedra is between 6.4 ° and 4.9 ° and the bonding angle is within the range 167 - 170 °. The decreasing trend of the tilting angle and the increment of the bonding angle as the Sr content increases can be understood due to the different atomic radii of Sr and La elements. The higher the Sr doping, the closer to unity is the tolerance factor and therefore

the closer the structures are to the ideal cubic LSMO (with no tilting and totally aligned octahedra). The reduction of the Mn-O and Mn-Mn distance is in accordance with the smaller cell volume due to a higher Sr content. Octahedral distortions present no significant changes with GGA + U.

An analysis of the electronic structure reveals a half-metallic behaviour for the ferromagnetic ground state, shown in Fig. S2a). Conduction states are composed of degenerate Mn e_g and O 2p levels. In the spin down channel, there is a pseudo-bandgap that decreases monotonically with the doping content as shown in Fig. S2c). Sr and La levels are considerably higher in energy, more than 4 eV above the Fermi level. Figure S2b) shows the PDOS adding a Hubbard term with U = 4.5 eV. Resemblance with bare GGA treatment are significant, but it is worth mentioning that the $t_{2g}^{\uparrow\downarrow}$ energy splitting increases. Oxygen levels are significantly modified in the spin up channel due to the coupling with the Mn t_{2g} levels. The pseudo-bandgap energy (E_g) follows a linear dependence as a function of the U parameter, as can be seen in Fig. S2d). Experimental work is included for comparison, pointing towards the known minor DFT underestimation of E_g. Better agreement is obtained for U $\sim$ 0.8 eV. Urushibara *et al.*[5] have reported a decrease in the magnetic saturation per Mn atom with the amount of Sr content, namely 3.5 μ_B/Mn for x = 0.3 and 3.4 μ_B/Mn for x = 0.4. The same trend is observed in our results (for both for GGA and GGA + U) with the best agreement with experiments being obtained for GGA (see Table 1). Inclusion of U = 4.5 eV increases the spin polarization by 0.2 - 0.3 μ_B.

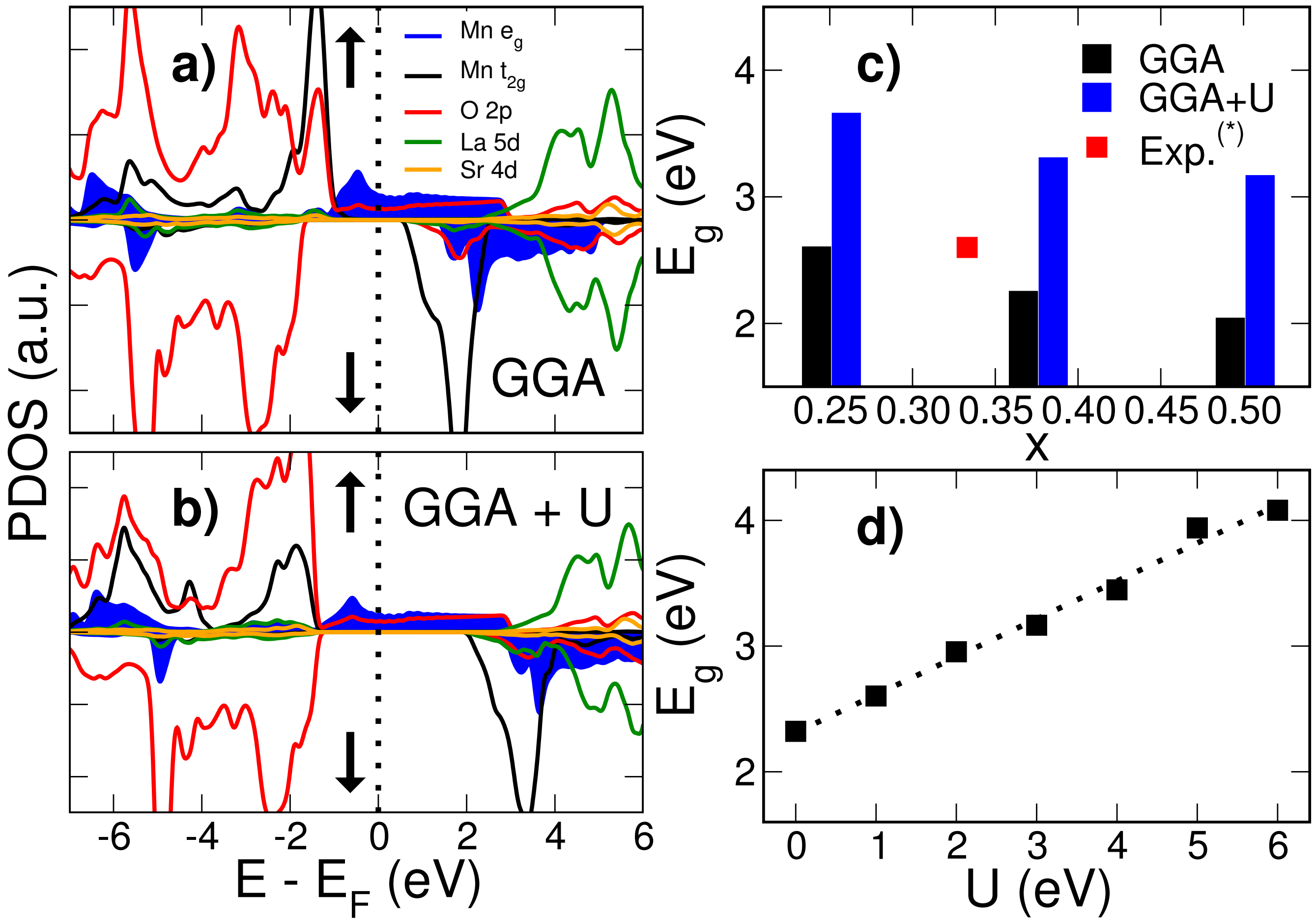

Figure S2. Electronic structure of pristine LSMO under GGA and GGA + U schemes. a) PDOS of LSMO (x = 0.375) with GGA, b) idem for GGA + U (4.5 eV). Spin up/down is drawn upwards/downwards (denoted by black arrows) and the Fermi energy marked with a dotted line. c) Pseudo-bandgap energy (E_g) as a function of Sr doping. (*) Ref.[6]. d) E_g versus U (with the linear trend in dotted lines).

Reduced LSMO: structural properties

Fig. S3 shows the reduced equilibrium lattice parameter for each of the three compositions with both local magnetic configurations (FM and SF) for bare GGA and GGA + U (with U = 4.5 eV). Reference pristine LSMO equilibrium lattice parameters are

included revealing, for x = 0.25 bare GGA in the FM magnetic state, a compression of the lattice as a result of incorporating oxygen vacancies, in clear controversy with the previously reported trends. By increasing the Sr concentration, this behaviour changes to an expansion of the lattice. The higher the Sr doping, the bigger the expansion. The reason for this phenomenon is the bonding defect state emerging as a result of introducing oxygen vacancies which in turn produces that the two Mn atoms that are nearest neighbors of the vacancy, get closer to each other as a result of atomic relaxation. Sr doping is equivalent to hole doping, gradually depopulating the defect level as x increases. For x = 0.5 this defect level is completely empty with no bonding effect. Introducing a Hubbard term provokes the defect level to become empty for the whole composition range. Therefore, we consistently obtain an expansion of the lattice which remains practically constant as a function of Sr doping. FS are employed to force an occupied/unoccupied defect state in the FM configuration. When the defect level is empty, there is an expansive effect. If the constrain populates the defect level, the decrease of the interatomic Mn-Mn distance (leading to a volume cell contraction for low doping) is compensated for higher doping, due to the displacements of the Sr atoms. For the SF case, an expansion of the lattice is found for all doping concentrations. This increase is rather small for bare GGA, while the expansion increments proportionally to the U value parameter.

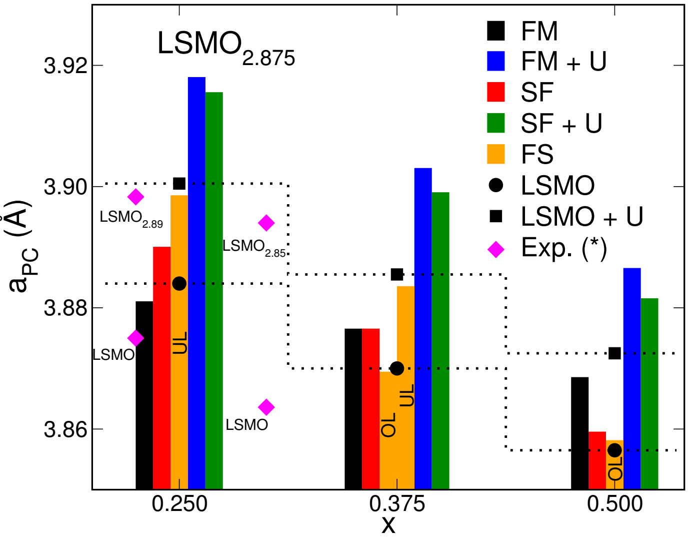

Figure S3. Reduced LSMO lattice pseudocubic parameter as a function of doping in the different magnetic configurations (FM and SF) for GGA (GGA + U when indicated). Constrained magnetic moment calculations (FS) are also plotted. Dotted lines are a guide to the eye for the pristine LSMO values. (*) Ref.[2].

References

1. Trukhanov, S. V. *et al.* Concentration-dependent structural transition in the $La_{0.70}Sr_{0.30}MnO_{(3-\delta)}$ system. *JETP Lett.* **84**, 254–257, DOI: 10.1134/S002136400617005X (2006).

2. Trukhanov, S. V. *et al.* Influence of oxygen vacancies on the magnetic and electrical properties of $La_{(1-x)}Sr_xMnO_{3-x/2}$ manganites. *The Eur. Phys. J. B - Condens. Matter Complex Syst.* **42**, 51–61, DOI: 10.1140/epjb/e2004-00357-8 (2004).

3. Martin, M. C. *et al.* Magnetism and structural distortion in the $La_{0.7}Sr_{0.3}MnO_3$ metallic ferromagnet. *Phys. Rev. B* **53**, 14285–14290, DOI: 10.1103/PhysRevB.53.14285 (1996).

4. Hemberger, J. *et al.* Structural, magnetic, and electrical properties of single-crystalline $La_{(1-x)}Sr_xMnO_3$ ($0.4 < x < 0.85$). *Phys. Rev. B* **66**, 094410, DOI: 10.1103/PhysRevB.66.094410 (2002).

5. Urushibara, A. *et al.* Insulator-metal transition and giant magnetoresistance in $La_{(1-x)}Sr_xMnO_3$. *Phys. Rev. B* **51**, 14103–14109, DOI: 10.1103/PhysRevB.51.14103 (1995).

6. Cheng, S. L., Du, C. H., Chuang, T. H. & Lin, J. G. Atomic replacement effects on the band structure of doped perovskite thin films. *Sci. Reports* **9**, 7828, DOI: 10.1038/s41598-019-44104-7 (2019).
